# Cellular angiofibroma of the inguinal scrotal region: A rare case report

**DOI:** 10.1097/MD.0000000000045504

**Published:** 2025-10-24

**Authors:** Shanshan He, Dasheng Qiu, Xiaoyan Hu, Lei Li, Nana Luo

**Affiliations:** a Department of Nuclear medicine, Hubei Cancer Hospital, Tongji Medical College, Huazhong University of Science and Technology, Wuhan, China.

**Keywords:** cellular angiofibroma, inguinal scrotal region, pathologic diagnosis

## Abstract

**Rationale:**

Cellular angiofibroma (CAF) is a rare benign mesenchymal tumor, which is difficult to diagnose due to its lack of specificity in clinical and imaging manifestations. This case highlights the challenges in the preoperative diagnosis of inguinoscrotal CAF and emphasizes the necessity of histopathologic confirmation.

**Patient concerns:**

A 53-year-old male was found to have a mass in the right inguinal area for 2 years, which had consciously increased for over a month. During the course of the disease, the patient did not experience any significant discomfort.

**Diagnoses:**

Physical examination showed a mass in the right inguinal area, about 14 cm in length, with a tough texture and good mobility. Imaging examinations (including ultrasound, computed tomography, and magnetic resonance imaging) have confirmed the presence of a mass in the right inguinal region, adjacent to the right spermatic cord. A benign tumor was suspected based on the clinical symptoms and imaging findings.

**Interventions:**

The patient underwent a complete surgical resection of the tumor under intravenous anesthesia. The tumor is gray–yellow in color, with a capsule, and can be separated from surrounding tissues.

**Outcomes:**

The surgery was successfully completed without any special discomfort to the patient, and the final pathological diagnosis was CAF. At 3 and 6 months, ultrasound examination showed no signs of tumor recurrence.

**Lessons:**

Accurate histopathological examination and long-term follow-up are key to ensuring a good prognosis for CAF patients.

## 1. Introduction

Cellular angiofibroma (CAF) is a rare benign mesenchymal tumor of the external genital tract and inguinal region of both sexes, which has been recognized only in recent years.^[1]^ CAF usually presents as a gradually increasing, solid, and painless mass, lacking specific imaging features, and is easily misdiagnosed as inguinal hernia, lipoma, and other diseases in clinical practice. Pathological diagnosis is the gold standard, and its differentiation from other diseases is not only based on morphological features but also on immunohistochemical features. It is crucial to establish the diagnosis because different lesions have different prognoses and treatments. CAF is treated by surgical resection and has a good prognosis, but a few cases of localized recurrence have been reported, so long-term follow-up is needed. This article summarizes the clinical features, pathological characteristics, imaging diagnosis and treatment, and prognosis of CAF, aiming to enhance the understanding of CAF and improve the diagnosis and treatment level of this tumor.

## 2. Case report

**Figure 1.**
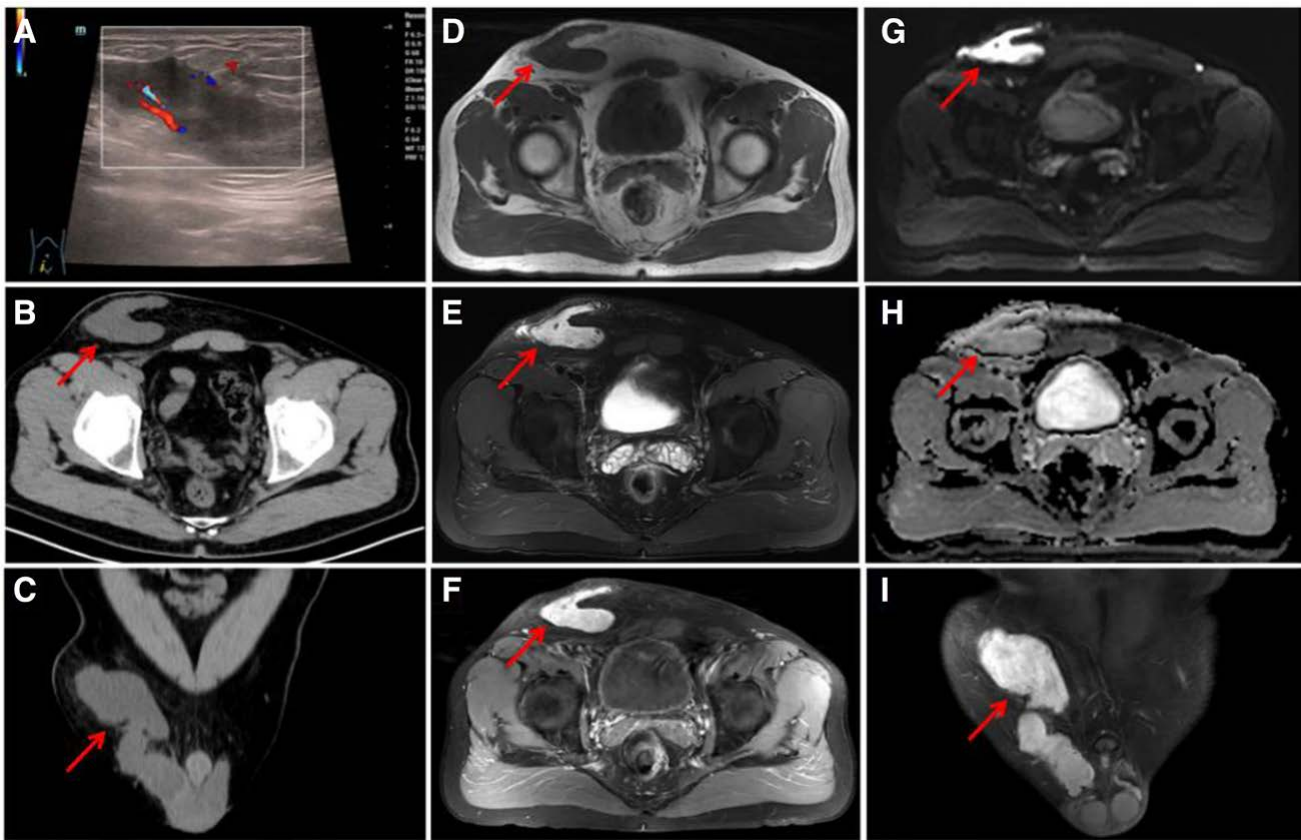
Patient scans. The lesion is indicated by the red arrow. (A) Ultrasonography showed a hyperechoic mass (15.0 × 6.1cm) in the right inguinal region with irregular morphology, with clear borders, and a small amount of blood flow signal could be seen within it. (B and C) Pelvic CT showed a soft tissue density mass (15.3 × 6.1cm) in the right inguinal region with irregular morphology and clear borders. (D–I) Pelvic MRI showed an irregular isotropic T1 long/slightly long T2 signal soft tissue mass (15.5 × 6.0cm) shadow in the right inguinal region, part of the level seemed to be connected with the right peritesticular adnexa, enhancement was visible, DWI was high signal, and ADC signal was not low. ADC = apparent diffusion coefficient, CT = computed tomography, MRI = magnetic resonance imaging.

The patient is a 53-year-old male who discovered a lump in the right inguinal region for 2 years, which was consciously enlarged for over a month. Physical examination showed that the right inguinal region subcutaneous palpable size of about 14 cm × 6 cm mass. It is lobulated, tough, and has good mobility. It does not change with body position, and there is no significant movement of the lump when pulling the testicle. Ultrasonography (Figure 1A) and pelvic computed tomography (CT; Figure 1B and C) all showed a mass in the right inguinal region with irregular morphology and clear borders. Pelvic magnetic resonance imaging (MRI; Figure 1D–I) showed an T1 long/slightly long T2 signal soft tissue mass, which appears to be adjacent to the right spermatic cord.

Under general anesthesia, complete resection of the right inguinal area mass was performed. The visible size of the tumor is ~16.0 × 9.0 × 4.5 cm, with a grayish yellow section and a capsule, and the boundary with surrounding tissues is still clear.

**Figure 2.**
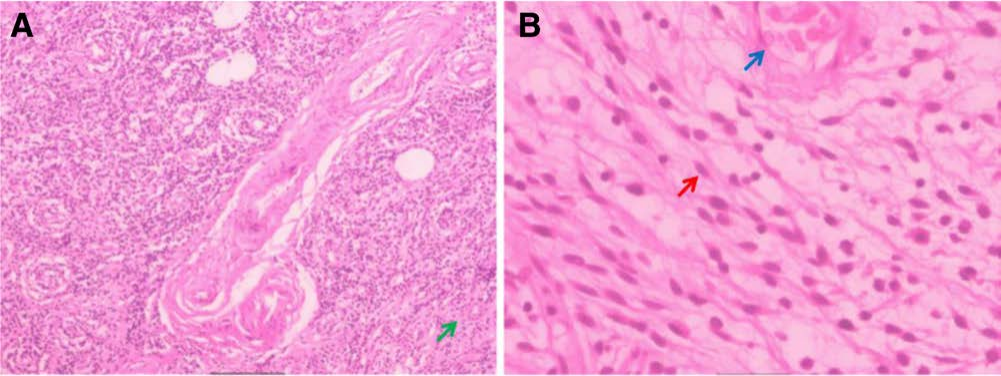
Pathological findings. (A) Low magnification and (B) high magnification show that tumor cells (as shown by the red arrow) are mainly short spindle shaped to oval shaped, with eosinophilic cytoplasm. The tumor cells are scattered and arranged, and some areas have abundant mucinous material in the stroma (as shown by the green arrow). There are a large number of thick-walled blood vessels in the stroma (as shown by the blue arrow), and some blood vessel walls are accompanied by glassy changes.

The specimen (Figure 2A and B) shows diffuse distribution of spindle-shaped cells, differentiated into dispersed fibrous vascular tissue and loose extracellular matrix. Immunohistochemistry (IHC) showed diffuse positivity for CD34 and vimentin, Rb protein is positive, epithelial membrane antigen is weakly positive, while estrogen receptor, S-100 protein, desmin, and smooth muscle actin (SMA) are negative. Based on this, the patient was finally diagnosed with CAF in the right inguinal scrotal area.

The patient recovered well after surgery without any obvious discomfort. Ultrasound examinations at 3 and 6 months after surgery did not indicate tumor recurrence.

## 3. Discussion

In addition to the distal reproductive tract, CAF can also occur in the hypopharynx, arms, rectum, and other sites.^[2–4]^ It is typically seen in middle-aged and older individuals, with a higher incidence in women and an earlier onset age than in men. Currently, the etiology and pathogenesis of CAF remain unclear. Some studies suggest that long-term estrogen therapy may be associated with the development of CAF.^[5,6]^ Others propose that it originates from perivascular stem cells with the potential to differentiate into adipose and myofibroblast tissue.^[7,8]^ CAF typically presents as spindle cells, small and medium-sized clear vessels, fine collagen, and mucoid stroma.^[9]^ IHC shows that tumor cells are diffusely positive for vimentin, and CD34 is positive in most cases, but desmin, S-100, actin, and epithelial membrane antigen are negative,^[1,10]^ which is consistent with the pathological features of the case we reported.

The ultrasound examination shows a well-defined heterogeneous or cystic mass with little or no obvious blood flow signal, which is consistent with the performance of our case. On plain CT scan, it usually presents as a well-defined soft tissue density nodule or mass with relatively uniform density and shows mild progressive enhancement on enhanced scan. In our case, CT plain scan shows similar findings, but it is impossible to assess its blood supply status due to the lack of enhancement. On MRI, the lesion has clear margins, appears as slightly higher signal on T2-weighted imaging (T2WI), and exhibits a signal intensity similar to that of muscle on T1-weighted imaging. Enhancement is heterogeneous, which may be related to the presence of abundant mucinous stroma and tumor vasculature.^[11,12]^ In clinical practice, diffusion-weighted imaging (DWI) with high signal intensity and limited diffusion, as well as significantly reduced apparent diffusion coefficient (ADC) values, usually indicates malignant lesions due to their dense cell density and limited diffusion of water molecules; on the contrary, benign lesions are often characterized by high ADC values. DWI combined with conventional MRI has high accuracy in distinguishing normal, benign, and malignant tumors.^[13,14]^ With our report, T2WI shows slightly higher/hyperintense signal, with uneven enhancement, and DWI shows high signal. These imaging findings are consistent with the benign nature of the lesion, as indicated by the non-decreased ADC signal.

However, traditional CT and MRI examinations lack specificity. Pathology, or more precisely, immunohistochemical analysis, is the gold standard for diagnosing this disease. Based on clinical features, imaging, and pathological manifestations, CAF needs to be differentiated from the following tumors: Spindle Cell Lipoma: It is a benign adipocytotic tumor with well-defined borders and intact periphery. Most patients are males in their 40s and 60s, and it occurs mainly in the nape of the neck, shoulders, and upper back. It is usually associated with chromosome 13 and/or chromosome 16 deletions. Tumor tissue is composed of different proportions of mature adipocytes, fibrous tissue, mucus stroma, and spindle cells. IHC is positive for CD34 in spindle cells and S-100 in mature adipocytes, both are negative for Rb protein. Density is slightly higher than subcutaneous fat and slightly lower than skeletal muscle on CT, enhancement of the nonfat component is markedly enhanced, MRI signal is dependent on the relative amount of fat, and positron emission tomography/CT tends to show high metabolic activity. Angiofibroblastoma: It is a well-defined benign myofibroblastic tumor rich in blood vessels. The tumor is small (<4cm) and most commonly occurs in the vulva of middle-aged and elderly women. The tumor tissue consists of alternating cell-rich and cell-sparse areas, with an abundance of small to medium-sized thin-walled blood vessels, and the tumor cells are distributed around the blood vessels. Tumor cells are strongly positive for Desmin and rarely express CD34 and SMA. CT enhancement scans often show moderate or significant enhancement, while T2WI shows low signal, unlike CAF. Solitary Fibrous Tumor: a rare benign spindle cell tumor, most commonly found in the visceral pleura. The tumor presents with unpatterned spindle cell proliferation, alternating high and low cellular areas, unlike CAFs where cells are evenly distributed. The blood vessels have a hemangiopericytomatous structure, and tumor cells express STAT6 but not SMA, estrogen receptor, or progesterone receptor. On CT, small tumors show uniform density, while larger tumors may show necrosis or cystic changes. Larger tumors present with “map-like” or progressive enhancement. Aggressive angiomyxoma: A rare, low-grade malignant mesenchymal tumor with infiltrative borders and a high local recurrence rate, commonly occurring in the female pelvis and vulva. Tumor size varies from 5 to 26 cm, and the tumor may lack a capsule or have a partial capsule. It is composed of tumor cells sparsely distributed in a lightly stained mucinous background, along with a rich fibrovascular stroma. Most tumor cells are spindle-shaped, and the immunophenotype is positive for SMA, Desmin, and CD34. Due to the mucinous matrix and fibrovascular bundles exhibiting spiral changes, both CT and MRI can show characteristic “whirlpool” and “lamellar” patterns.

The recommended strategy is complete resection with negative surgical margins. Most cases of CAF have a favorable prognosis, with low recurrence and no potential for metastasis. In some cases, an abrupt transition to areas of sarcomatous transformation may occur, but up to now, we are not aware of any cases of tumor metastasis with sarcoma characteristics, indicating that sarcoma does not necessarily confer invasive biological behavior on CAF.^[15]^ However, according to reports, a few cases have experienced local recurrence.^[16]^

Effective prognostic stratification is crucial for optimizing the treatment of cancer patients. Among many common malignant tumors, prognostic models based on systemic inflammatory markers (such as the modified Glasgow prognostic score) or comprehensive hematological parameters (such as absolute monocyte count, albumin-to-globulin ratio) have been fully validated.^[17–19]^ These tools greatly assist clinical decision-making. In contrast, due to the extremely low incidence rate, it is a great challenge to establish similar evidence-based prognostic tools in the field of CAF. This gap makes precise histopathological diagnosis and strict, long-term postoperative follow-up play an irreplaceable role in the management of CAF. The findings of this study reinforce the notion that in the absence of sensitive biomarkers, clinical vigilance, and attention to diagnostic details are key to early detection of problems and improvement of patient prognosis.

## 4. Conclusion

The diagnosis of CAF is a complex process from clinical suspicion, imaging findings to pathological diagnosis. Emphasis is placed on radical surgical resection in treatment. Due to the lack of prognostic prediction tools, accurate histopathological examination and long-term follow-up are the cornerstones to ensure good outcomes for patients.

## Acknowledgments

We would like to thank all the staff and nurses for their kind cooperation. We would also like to thank the patient.

## Author contributions

**Conceptualization:** Dasheng Qiu.

** Writing – original draft:** Shanshan He, Nana Luo.

**Writing – review & editing:** Xiaoyan Hu, Lei Li, Nana Luo.
